# Effect of P to A Mutation of the N-Terminal Residue Adjacent to the Rgd Motif on Rhodostomin: Importance of Dynamics in Integrin Recognition

**DOI:** 10.1371/journal.pone.0028833

**Published:** 2012-01-04

**Authors:** Jia-Hau Shiu, Chiu-Yueh Chen, Yi-Chun Chen, Yao-Tsung Chang, Yung-Sheng Chang, Chun-Hao Huang, Woei-Jer Chuang

**Affiliations:** 1 Department of Biochemistry and Molecular Biology, Institute of Basic Medical Sciences, National Cheng Kung University College of Medicine, Tainan, Taiwan; 2 Institute of Biopharmaceutical Sciences, National Cheng Kung University College of Medicine, Tainan, Taiwan; University of South Florida College of Medicine, United States of America

## Abstract

Rhodostomin (Rho) is an RGD protein that specifically inhibits integrins. We found that Rho mutants with the P48A mutation 4.4–11.5 times more actively inhibited integrin α5β1. Structural analysis showed that they have a similar 3D conformation for the RGD loop. Docking analysis also showed no difference between their interactions with integrin α5β1. However, the backbone dynamics of RGD residues were different. The values of the *R_2_* relaxation parameter for Rho residues R49 and D51 were 39% and 54% higher than those of the P48A mutant, which caused differences in S^2^, R_ex_, and τ_e_. The S^2^ values of the P48A mutant residues R49, G50, and D51 were 29%, 14%, and 28% lower than those of Rho. The R_ex_ values of Rho residues R49 and D51 were 0.91 s^−1^ and 1.42 s^−1^; however, no R_ex_ was found for those of the P48A mutant. The τ_e_ values of Rho residues R49 and D51 were 9.5 and 5.1 times lower than those of P48A mutant. Mutational study showed that integrin α5β1 prefers its ligands to contain **(G/A)**RGD but not **P**RGD sequences for binding. These results demonstrate that the N-terminal proline residue adjacent to the RGD motif affect its function and dynamics, which suggests that the dynamic properties of the RGD motif may be important in Rho's interaction with integrin α5β1.

## Introduction

The tripeptide sequence Arg-Gly-Asp (RGD) is the consensus sequence of many adhesive proteins, such as fibronectin, fibrinogen, vitronectin, and von Willebrand factor [1], [2], [3]. In mammals, 18 α and 8 β subunits assemble into 24 integrins. The RGD sequence is recognized by half of the 24 known integrins, whereas alternative short peptide sequences are recognized by other integrins [4]. In addition to adhesive proteins, the RGD sequence is found in many proteins, including dendroaspin [5], decorsin [6], savignygrin [7], streptopain [8], γ-bungarotoxin [9], human herpesvirus 8 envelope glycoprotein B [10], and disintegrins [11]. Disintegrins are the peptides found in snake venoms of the viper family and mainly inhibit the functions of β1- and β3-associated integrins. They were first identified as inhibitors of integrin αIIbβ3 and were subsequently shown to bind with high affinity to other integrins and to block the interaction of integrins with RGD-containing proteins. They contain 47–84 amino acids with 4–7 disulfide bonds. The RGD or KGD sequences in this disintegrin family are the most important in recognizing the integrin αIIbβ3 [12], [13], [14], [15], [16]. Analyses of 3D disintegrin structures show that they consist of a series of tightly packed loops and turns held together by disulfide bonds [17], [18], [19], [20], [21]. The RGD motif is located at the apex of a 5–11 residue loop, between two β strands of the protein, protruding 10–17 Å from the protein core [13]. The R and D sidechains in a flexible loop do not interact but nearly oppose each other by 180°.

Many studies have shown that the residues flanking the RGD motif of RGD-containing proteins affect their binding specificities and affinities on integrins [7], [10], [22], [23], [24], [25]. For example, disintegrins with an ARGD**W** sequence have a higher affinity for binding with the integrin αIIbβ3, whereas disintegrins with an ARGD**N** sequence preferentially bind with integrins αvβ3 and α5β1 [24]. The amino acid sequences of the RGD loop from RIPRGDMP to TAVRGDGP of rhodostomin (Rho), resulting in a 196-fold decrease in inhibiting integrin αIIbβ3 [9]. Replacement of the N-terminal alanine with the proline of the RGD motif of elagantin, a disintegrin with an **A**RGDMP sequence, diminishes its binding to integrin α5β1 [25], which suggests that replacing the N-terminal proline with the alanine of the RGD motif may increase its binding to integrin α5β1. Therefore, it is of interest to study the effect of the N-terminal proline or alanine residue adjacent to the RGD motif on the function, structure, and dynamic relationships of disintegrin.

In this study, we used Rho as the model protein to investigate the effect of the N-terminal proline residue adjacent to the RGD motif on the dynamics of disintegrin and the structure-activity relationships of RGD-containing proteins. Rho is obtained from *Calloselasma rhodostoma* venom and belongs to the family of disintegrins [26], [27], [28]. It consists of 68 amino acids, including 12 residues of cysteine and a PRGDMP sequence at positions 48–53. We previously showed that Rho expressed in *Pichia pastoris* (*P. pastoris)* has the same function and structure as native protein [28]. In the present study, we expressed Rho P48A mutants and determined their activities in inhibiting the integrins αIIbβ3, αvβ3, and α5β1. We also used nuclear magnetic resonance (NMR) spectroscopy to compare 3D structures and backbone dynamics.

## Materials and Methods

### Expression of Rho and its Mutants in P. pastoris and Purification

The expression of Rho and eleven mutants (P48A, M52W, P48A/M52W, M52N, P48A/M52N, M52G/P53W, P48A/M52G/P53W, M52D/P53L, P48A/M52D/P53L, M52D/P53M, and P48A/M52D/P53M) in *P. pastoris* was accomplished by following protocols previously described [27], [28]. The expression kit and the yeast transfer vector, pPICZαA, were purchased from Invitrogen. The wild-type construct was used to produce the mutations using overlap extension PCR. The construct was transformed into the *Pichia* strain, X33, using a *Pichia* EasyComp kit from Invitrogen.

Unlabelled and ^15^N-labelled Rho and its mutants were produced by following protocols previously described [27], [28]. The unlabelled proteins were produced as follows: 100 µL of cell stock grew at 30°C in 100 mL of yeast nitrogen base (YNB) medium (1% yeast extract, 2% peptone, and 2% dextrose) containing 100 µg/mL of Zeocin for 48 h. Cells were then transferred into 900 mL of YNB medium. After another 48 h, the cells were collected by centrifugation and grown in 1 L of minimal methanol medium (1.34% YNB with ammonium sulphate without amino acids and 4×10^−5^% biotin). Methanol (1% w/v) was added once every 12 h to induce protein expression for 2 days. The ^15^N-labelled proteins were produced as follows: 100 µL of cell stock grew at 30°C in 100 mL of ^15^N minimal medium (0.34% YNB without ammonium sulphate and amino acids, 2% dextrose, and 0.05% ^15^NH_4_Cl) in 100 mM of potassium phosphate buffer with 100 µg/mL of Zeocin for 48 h. The cells were then transferred into 900 mL of ^15^N minimal medium. After another 24 h, the cells were collected by centrifugation and grown in 1 L of ^15^N minimal medium in 100 mM of potassium phosphate buffer with 4×10^−5^% biotin. The methanol in the medium was maintained at 1% (w/v) in order to induce protein expression for 48 h.

The supernatant was collected by centrifugation and dialyzed twice against 10 L of H_2_O and once against 5 L of binding buffer (50 mM Tris-HCl buffer at pH 8.0). The dialyzed solution was loaded into a Ni^2+^-chelating column and proteins were eluted using elution buffer containing 200 mM of imidazole. Proteins were then purified using C18 reversed-phase HPLC with a gradient of 20–30% acetonitrile. The recombinant proteins were more than 95% pure, as determined using tricine-SDS-PAGE.

### Fibronectin Purification

Fibronectin was purified from citrated human plasma using gelatin-Sepharose 4B affinity chromatography as previously described [29]. One hundred millilitres of human plasma was centrifuged at 5000 rpm for 30 min and then filtered through Whatman filter paper. The filtrate was applied to a pre-equilibrated gelatin-Sepharose resin with phosphate-buffered saline (PBS: 10 mM phosphate buffer, 0.15 M NaCl [pH 7.4]) at pH 7 containing 5 mM EDTA, 0.05% (w/v) NaN_3_, and 1 mM benzamidine. The resin was washed with 1 M NaCl and 1 mM benzamidine at pH 7, and fibronectin then was eluted using 1 M urea and 1 mM benzamidine at pH 7. The fractions were dialyzed three times against 4 L of PBS buffer at pH 7.4 and concentrated using Amicon with a 10-kDa cutoff membrane. The yields of fibronectin were 15–20 mg, and the purification of human fibronectin was greater than 95% as determined using SDS-PAGE. Purified fibronectin was stored at −70°C until it was used.

### Mass Spectrometric Measurements

The molecular weights of Rho mutant proteins were confirmed using an API 365 triple quadrupole mass spectrometer equipped with a TurboIonSpray source (PE-Sciex, Thornill, Canada). Protein solutions (1–10 µM in 50–90% methanol or acetonitrile with 0.1% formic acid) were infused into the mass spectrometer using a syringe pump (Harvard Apparatus, South Natick, MA, USA) at a flow rate of 12–20 µL/min to acquire full-scan mass spectra. The electrospray voltage at the spraying needle was optimized at 5000–5300 V. The molecular weights of proteins were calculated using computer software provided with the API 365 mass spectrometer.

### Platelet Aggregation Assay

Venous blood (9 parts) from healthy donors who had not received any medication for at least two weeks were collected in 3.8% sodium citrate (1 part). Blood was centrifuged at 70× *g* for 10 min to obtain platelet-rich plasma (PRP) and allowed to stand for 5 min. Then, PRP was collected. Platelet-poor plasma (PPP) was prepared from the remaining blood by centrifuging it at 800× *g* for 10 min. The PPP platelet count was measured on a haematology analyzer, and the platelets were diluted to 250,000 platelets/µL. A solution of PRP (190 µL) and either Rho or PBS buffer (10 µL) was incubated for 5 min in a Hema Tracer 601 aggregometer at 37°C. Ten microlitres of 200 mM ADP was added to monitor the response of platelet aggregation by light transmission.

### Cell Adhesion Assay

A cell adhesion assay was done using protocols previously described [29]. Ninety-six-well microtitre plates (Costar, Corning, USA) were coated with 100 µL of PBS buffer containing 200 µg/ml fibrinogen or 25 µg/mL fibronectin, and incubated overnight at 4°C. Non-specific protein binding sites were blocked by incubating each well with 200 µL of heat-denatured 1% bovine serum albumin (BSA) (Calbiochem) at room temperature for 1.5 h. The heat-denatured BSA was discarded and each well was washed twice with 200 µL of PBS.

Chinese hamster ovary (CHO) cells that expressed the integrins αvβ3 (CHO-αvβ3) and αIIbβ3 (CHO-αIIbβ3) were kindly provided by Dr. Y. Takada (Scripps Research Institute) and maintained in Dulbecco's Modified Eagle's Medium (DMEM) medium [29]. Human erythroleukemia K562 cells were purchased from ATCC and cultured in Roswell Park Memorial Institute (RPMI)-1640 medium containing 5% foetal calf serum. Harvested K562 cells were washed in PBS buffer containing 1 mM EDTA and resuspended in Tyrode's buffer (150 mM NaCl, 5 mM KCl, and 10 mM Hepes) at pH 7.35 containing 1 mM MgSO_4_, 2 mM CaCl_2_, and 500 µM MnCl_2_. CHO and human erythroleukemia K562 cells were diluted to 3 and 2.5×10^5^ cells/mL, respectively, and 100 µL of the cells were used for the assay. Rho and its mutants (0.001–500 µM), which were used as inhibitors, were added to the cells and incubated at 37°C in a 5% CO_2_ atmosphere for 15 min. The treated cells were then added to the coated plate and reacted at 37°C (5% CO_2_) for 1 h. The reacting solution was then discarded and non-adhered cells were removed by washing them twice with 200 µL of PBS. After the non-adhered cells had been removed by rinsing the wells with the same buffer, adhered cells were quantified using a crystal violet assay. The well was fixed with 100 µL of 10% formalin for 10 min and then dried. A solution of 50 µL of 0.05% crystal violet was added to the well at room temperature for 20 min. Each well was then washed four times with 200 µL of distilled water and dried. Colorization was done by adding 150 µL of colorizing solution (50% alcohol and 0.1% acetic acid). The resulting absorbance was read at 600 nm and the readings were correlated with the number of adhering cells. Inhibition was defined as
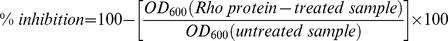
The reported IC_50_ values are the average of at least three separate experiments.

### Nuclear Magnetic Resonance (NMR) Spectroscopy

NMR experiments were done at 27°C on a Bruker Avance 600- and 700-MHz spectrometer equipped with pulse field gradients and xyz-gradient triple-resonance probes. In these experiments, samples were dissolved in 10% D_2_O/90% H_2_O or 100% D_2_O at a concentration of 3 mM; pH was adjusted with 100 mM KOD to 6.0. The data were processed with Topspin Version 1.3 software and analyzed with Aurelia software. 2D NOESY, TOCSY, and DQF-COSY NMR spectra were recorded in the phase-sensitive absorption mode with quadrature detection in both F1 and F2 dimensions. A concentration of 2 mM ^15^N labeled Rho and its P48A mutant was used for the 2D ^1^H-^15^N HSQC, 3D ^1^H-^15^N edited-TOCSY-, and NOESY-HSQC experiments. Mixing times of 30–90 ms and 60–150 ms were used for TOCSY and NOESY experiments, respectively. The centre frequencies of double resonance experiments were 4.75 ppm (^1^H) and 118 ppm (^15^N). The observed ^1^H chemical shifts were referenced with respect to the H_2_O or HOD signal, which was 4.754 ppm downfield from external sodium 3-trimethylsilylpropionate-2,2,3,3-d4 (TSP) in D_2_O (0.0 ppm) at 300°K. The nitrogen chemical shift was referenced to external ^15^NH_4_Cl (3 mM in 1 M HCl) at 300°K, which is 24.93 ppm downfield from liquid NH_3_.

### Structure Calculations

Structures were calculated using the program X-PLOR with the hybrid distance geometry-dynamical simulated annealing method [30]. NOESY cross-peak intensities—categorized into strong, medium, weak, and very weak—were converted into distance constraints of 1.8–2.8, 1.8–3.6, 1.8–5.0, and 2.5–6.0 Å, respectively. Pseudoatom corrections were used for methylene, methyl, and aromatic protons, and an additional 0.5 Å was added to the upper limit distances involving methyl protons. The dihedral angles ϕ were determined from the ^3^J_NHα_ coupling constants. For ^3^J_NHα_ values less than 5 Hz, ϕ values were restricted from −30° to −90°, and for ^3^J_NHα_ values greater than 10 Hz, ϕ values were restricted from −100° to −170°. Two restraints were used for each NH-CO backbone hydrogen bond with d_N-O_ restricted to 2.4–3.3 Å and d _H-O_ to 1.7–2.3 Å. A family of 100 structures was generated using NOE distance, dihedral angle, and hydrogen bond restraints. The S-S covalent bonds were deleted and reintroduced as pseudo-NOE distances with the S-S distances constrained to the upper limit of 2.1 Å. During the first phase of dynamics at 2000°K, the value of the force constant of the NOE term was kept constant at 50 kcal/mol^−1^ Å^−2^. The repulsion term was gradually increased from 0.03 to 4.0 kcal/mol^−1^ Å^−2^, and the torsion angle term from 5 to 200 kcal/mol^−1^rad^−2^. The simulated annealing refinement consisted of a 9-ps cooling dynamic followed by 200 cycles of Powell minimization. The twenty lowest-energy structures were accepted based on violations of distance restraints less than 0.5 Å, dihedral angle restraints less than 5°, a van der Waals energy cutoff value of 35 kcal/mol, and an NOE energy cutoff value of 55 kcal/mol. The structure figures were prepared using the MOLMOL or the PyMOL program [31], [32].

### Measurements of NMR Dynamics

Backbone dynamics of Rho and its P48A mutant were studied by two-dimensional proton-detected heteronuclear NMR spectroscopy. The ^15^N- spin-lattice (*R_1_*) and spin-spin (*R_2_*) relaxation rate constants and steady-state ^1^H-^15^N NOEs were measured from ^1^H-detected ^1^H-^15^N correlation spectra recorded with sensitivity-enhanced pulse sequences. A recycle delay of 6 s was used, and 128 complex T_1_ increments of 32 scans were acquired. A series of 10 experiments with relaxation delays of 30, 100, 150, 300, 450, 600, 800, 1000, 1500, and 3000 ms were done to measure T_1_. A series of 10 experiments with relaxation delays of 18, 36, 48, 72, 90, 100, 120, 150, 300 and 500 ms were done to measure T_2_. The longitudinal and transverse relaxation rate constants, *R_1_* and *R_2_*, were obtained from exponential fits of the peak height data using least-squares fit software SigmaPlot (Jandel Scientific). The reported *R_i_* values are the mean values of two independent data sets. In the NOE experiment, two spectra—one with the NOE and one without—were collected. The NOE was calculated as the ratio of peak heights in spectra collected with and without NOE. The reported NOE value was the average value of three pairs of NOE experiments.

The heteronuclear ^15^N relaxation rate constants, *R_1_* and *R_2_*, and the ^1^H-^15^N steady state NOE values were analyzed using the FastModelFree program [33]. In this approach, the overall and internal molecular motions were assumed to be independent, and the spectral density function for a molecule undergoing isotropic tumbling was calculated using the appropriate expression:
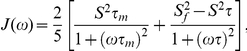
where 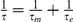 and 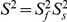, τ_m_ is the overall rotational correlation time of the molecule, τ_e_ is the effective correlation time for the motions on the slower of the two time scale, S^2^ is the square of the generalized order parameter, and S^2^_s_ and S^2^_f_ are the squares of order parameters for the motion on the slow and fast time scale, respectively [34].

### Molecular Docking

The dockings of Rho and its P48A mutant to integrin α5β1 were done using the docking program HADDOCK 2.1 with hydrogen bond and distance restraints [35]. The structure of integrin α5β1 was modelled using the program MODELLER [36] with integrin αvβ3 (PDB code 1L5G) as the template, and the starting structures of Rho and its P48A mutant were their average minimized NMR structures. The interaction restraints were derived from the X-ray structure of integrin αvβ3 in complex with a cyclic pentapeptide (c(-RGDf[NMe]V-)) using the software iMoltalk [37]. The defined distance threshold was 4 Å, and the interaction restraints between the RGD motif and integrin were used for calculation. The input restraints between the R49, G50, and D51 residues and integrin α5β1 were 30, 7, and 39, respectively. They were the contacts between the R49 residue and the residues F187, Q189, and D227 of integrin α5, between the G50 residue and the residues L214 and S216 of β1, and between the D51 residue and the residues S121, Y122, S123, G212, N213, and E218 and Mn^2+^ of the MIDAS (metal-ion-dependent adhesion site) of β1. Additional 0.5 Å distance was added to the upper and lower limits in the direct interaction restraints. Using these restraints, the standard HADDOCK protocol for protein docking was done with minor modifications. This protocol combines three stages of molecular dynamics calculations, including heating and cooling with a progressive increase of the flexibility at the binding interface. In the first stage, 500 conformations were calculated using a rigid-body docking protocol. The best 100 structures in terms of their inter-molecular energies were refined by semi-flexible simulated annealing in the second stage. Both the side-chains and the backbone atoms of the residues 46–54 were defined as flexible and allowed the residues to move in a semi-rigid-body docking protocol to search for conformational rearrangements. The resulting 100 structures with the lowest intermolecular energy values were refined with explicit water molecules in the last stage. The structures were classified by clustering based on the pairwise RMSD differences. The structures were found by fitting them over the RGD residues with an average RMSD value <1.5 Å for the backbone atoms of all the amino acids in 100 integrin complexes.

### Protein Data Bank Accession Number and Assignment

The coordinates of 20 calculated structures of P48A mutant were deposited in the Protein Data Bank under accession number 2PJI.

### Ethics Statements

The ethics approval for our study was approved by an independent ethics committee of National Cheng Kung University. The collection of human serum was followed the guidelines and regulation. We have obtained written informed consent from all participants involved in this study.

## Results

### Protein Expression and Purification of Rho and its Mutants

Rho and eleven mutants were expressed with pPICZαA vector in *P. pastoris* X33 strain. Rho and its P48A, M52W, P48A/M52W, M52N, P48A/M52N, M52G/P53W, P48A/M52G/P53W, M52D/P53L, P48A/M52D/P53L, M52D/P53M, and P48A/M52D/P53M mutants produced in *P. pastoris* were purified to homogeneity, according to SDS-polyacrylamide gel electrophoresis (data not shown), using Ni^2+^-chelating chromatography and C18 reversed-phase HPLC. The yields of Rho and its mutants were 10–25 mg/L. Mass spectrometry showed that the experimental molecular weights deviated less than 1 Da when compared with the theoretical values, which had been calculated by assuming that all cysteines form disulfide bonds, and would result in the formation of six disulfide bonds in Rho and its mutants (Table S1 and Figure S1).

### Inhibition of Platelet Aggregation

Rho produced in *P. pastoris* inhibited platelet aggregation with a K_I_ of 83.2±10.4 nM (Table 1), which is as potent as native Rho [21]. The mutation of P48 to A in Rho caused only a 1.3-fold decrease in activity in the inhibition of platelet aggregation with a K_I_ of 110.3±14.1 nM. These results showed that the P48 residue in the RGD loop of Rho has little effect on the interaction between Rho and platelet integrin αIIbβ3.

**Table 1:** Summary of inhibition of platelet aggregation and cell adhesion by Rho and its P48A mutant.

	IC50 (nM)^a^		
Integrin/Cell	Rho	P48A Mutant	IC50_Rho_/IC50_P48A_
αIIbβ3/Platelet^b^	83.2±10.4	110.3±14.1	0.75
αIIbβ3/CHO^c^	21.0±11.2	31.6±7.2	0.66
αvβ3/CHO^d^	13.0±5.7	15.8±3.3	0.82
α5β1/K562^e^	256.8±27.5	59.0±18.4	4.35

a The IC50 values are the average value of 3–5 experiments.

b Inhibition of ADP-induced platelet aggregation by Rho proteins was measured in human PRP.

c Inhibition of integrin αIIbβ3-expressing CHO cell adhesion to immobilized fibrinogen by Rho proteins.

d Inhibition of integrin αvβ3-expressing CHO cell adhesion to immobilized fibronectin by Rho proteins.

e Inhibition of K562 cell adhesion to immobilized fibronectin by Rho proteins.

### Inhibition of Cell Adhesion to Fibrinogen and Fibronectin

The adhesion of CHO cells that express αIIbβ3 and αvβ3 to immobilized fibrinogen is dependent on the affinity states of the αIIbβ3 and αvβ3 integrins, respectively [38]. These assays used mAbs that blocked the functions of αIIbβ3 and αvβ3 [25], [39]. In addition, the adhesion of K562 cells to fibronectin in the presence of 500 µM of Mn^2+^ is predomiantly α5β1-dependent [25]. Rho and its P48A mutant inhibited the adhesion of CHO cells that express the integrin αIIbβ3 to immobilized fibrinogen with the IC_50_ values of 21±11.2 and 31.6±7.2 nM, respectively (Table 1). This result was consistent with their activity in inhibiting ADP-induced platelet aggregation. Similarly, Rho and its P48A mutant inhibited the adhesion of CHO cells that express the integrin αvβ3 to immobilized fibrinogen with the IC_50_ values of 13±5.7 and 15.8±3.3 nM, respectively. Rho and its P48A mutant inhibited K562-cell adhesion to immobilized fibronectin with the IC_50_ values of 256.8±87.5 and 59.0±28.4 nM, respectively. In contrast to their inhibition of integrins αIIbβ3 and αvβ3, P48A mutant showed a 4.4-fold increase in its inhibition of integrin α5β1. These results showed that the N-terminal alanine residue adjacent to the RGD motif increased its inhibition of integrin α5β1, but not of integrins αIIbβ3 and αvβ3.

We also expressed a series of Rho proteins, M52W, P48A/M52W, M52N, P48A/M52N, M52G/P53W, P48A/M52G/P53W, M52D/P53L, P48A/M52D/P53L, M52D/P53M, and P48A/M52D/P53M mutants to confirm the effect of the N-terminal alanine of the RGD motif on inhibiting integrin α5β1. The results were consistent with that of P48A mutant, and the mutant proteins containing a P48A mutation showed a 4.4–11.5-fold increase in the inhibition of integrin α5β1 (Table 2). These results suggest that RGD proteins with the RGD motif containing an N-terminal proline residue may weaken their binding to integrin α5β1.

**Table 2:** Summary of inhibition of K562 cell adhesion to fibronectin by Rho and its mutants.

P-type Protein^a^						^P^IC50 (nM)	A-type Protein^b^						^A^IC50 (nM)	^P^IC50 (nM)/^A^IC50 (nM)
P	R	G	D	M	P	256.8±87.5	**A**	R	G	D	M	P	59.0±28.4	4.4
P	R	G	D	**W**	P	403.5±43.9	**A**	R	G	D	**W**	P	52.0±14.4	7.8
P	R	G	D	**N**	P	357.0±80.6	**A**	R	G	D	**N**	P	44.1±17.6	8.1
P	R	G	D	**G**	**W**	1238.4±632.9	**A**	R	G	D	**G**	**W**	190.8±75.9	6.5
P	R	G	D	**D**	**L**	3017.0±801.5	**A**	R	G	D	**D**	**L**	526.7±200.3	5.7
P	R	G	D	**D**	**M**	4047.3±1784.3	**A**	R	G	D	**D**	**M**	350.8±81.0	11.5

a P-type proteins contain a P residue at the 48 position.

b A-type proteins contain a A residue at the 48 position.

The mutated positions are shown in boldface.

### Structure Determination

The solution structure of Rho and the 3D structure of the P48A mutant were determined using NMR spectroscopy and the hybrid distance geometry-dynamical simulated annealing method [27]. NMR spectra were recorded at pH 6.0. NMR assignment of the P48A mutant was obtained by analyzing standard 2D homonuclear and 3D heteronuclear NMR data (data not shown). We also did NOESY experiments at pH 6.0 in 100% D_2_O to determine their six disulfide bonds. Their pairings were identified using CβH to CβH, CαH to CβH, and CαH to CαH NOEs between different cysteines [28]. Our analysis showed that the disulfide parings (C4–C19, C6–C14, C13–C36, C27–C33, C32–C57, and C45–C64) of Rho and its P48A mutant followed the flavoridin-type but not albolabrin-type pattern [18]. The secondary structures of the P48A mutant consisted of three short regions of two-stranded antiparallel β-sheets (residues 14–16 and 22–19, 32–34 and 37–39, and 43–45 and 55–57). The formations of three two-stranded antiparallel β-sheets were characterized by the CαH-CαH, CαH-NH, and NH-NH NOE patterns of the connecting strands, the slowly exchanging amide protons, and the downfield-shifted α protons. Three short regions of antiparallel β-sheet structures have been observed by the NOE pattern analysis (Figure S2).

**Figure 1:**
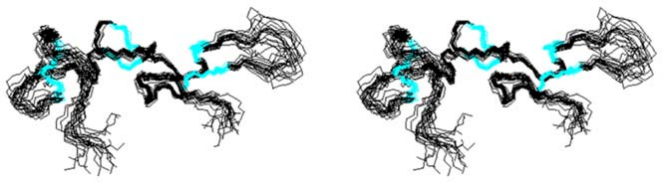
Structures of Rho P48A mutant. Stereo views of the 20 lowest-energy NMR structures of the Rho P48A mutant.

The 3D Structure of the P48A mutant was calculated using 1048 experimentally derived restraints with an average of 15.4 restraints per residue (Table 3). The 20 best structures of the P48A mutant from 100 initial structures are shown in Figure 1. The backbone RMSD value of the P48A mutant is 1.04±0.23 Å, and the backbone RMSD values of the P48A mutant for three β-sheet regions (13–14, 20–21, 33–34, 37–38, 43–45, and 55–57) was 0.38±0.10 Å. Based on a Ramachandran analysis, all dihedral angles of the P48A mutant were in the allowed region. A summary of the restraints and structural statistics is presented in Table 3. Overall, the tertiary structure of the P48A mutant has an elongated and asymmetric shape and consists of three two-stranded antiparallel β-sheets with many tight turns and loops.

**Table 3:** Summary of structural restraints and statistics for Rho P48A mutant.

Restraints used in the structure calculation	
Distance and dihedral angle restraints	
Intra-residue	145
Sequential	111
Medium range	333
Long range	387
Hydrogen bonds	9
Dihedral angles	57
Total	1048
Energy statistics	
X-PLOR energy (kcal mol^−1^)	
E_NOE_	23.38±6.91
E_vdw_	11.49±2.99
Geometric statistics	
Deviations from idealized geometry	
All backbone atoms (Å)	1.04±0.23
Backbone atoms (13–14, 20–21, 33–34, 37–38, 43–45, and 55–57) (Å)	0.38±0.10
All heavy atoms (Å)	1.62±0.25
Heavy atoms (13–14, 20–21, 33–34, 37–38, 43–45, and 55–57) (Å)	0.87±0.12
Ramachandran analysis	
Most favored regions (%)	75.2
Additionally allowed regions (%)	23.6
Generously allowed regions (%)	1.2

### No Structural Difference between Rho and its P48A Mutant

**Figure 2:**
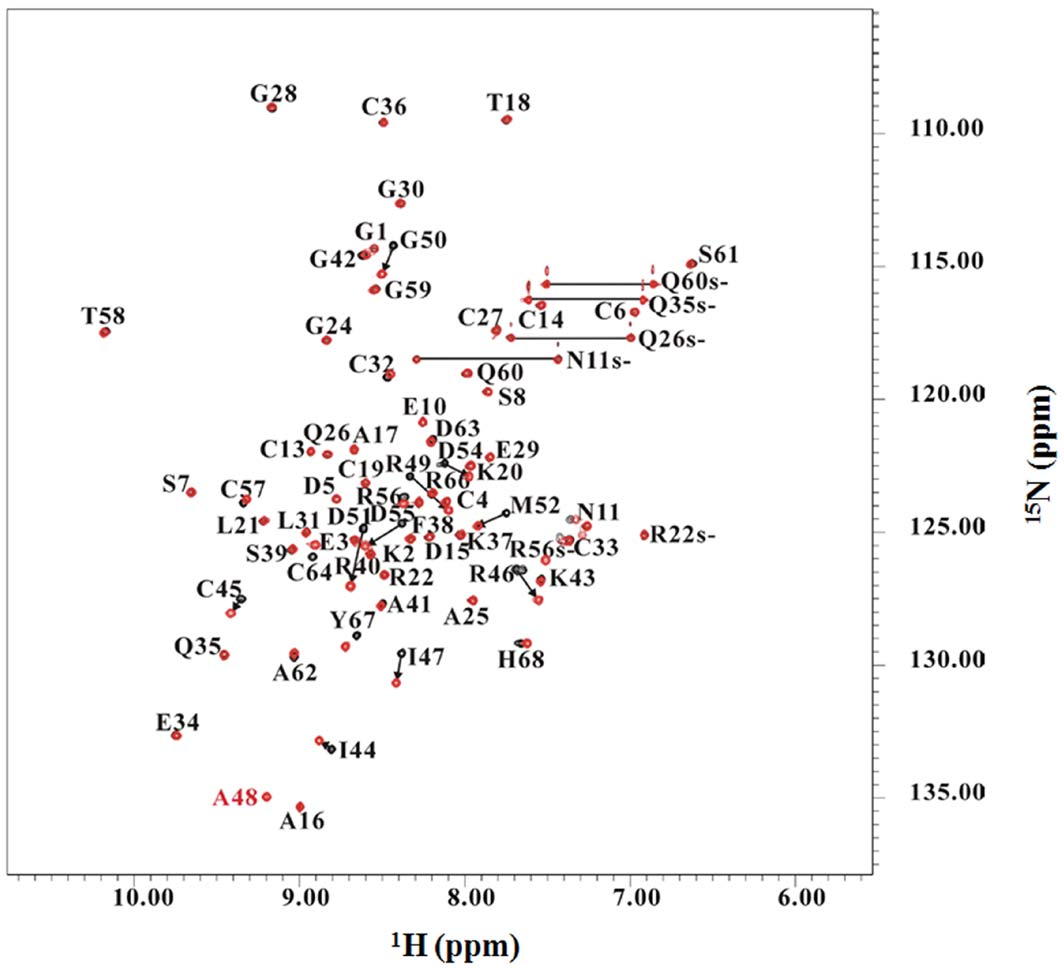
2D ^1^H-^15^N HSQC spectra of Rho (black) and its P48A mutant (red) at pH 6.0. Correlation peaks are labelled according to residue type and sequence number. The peaks connected by lines correspond to Gln and Asn side chain NH_2_ groups. The peaks denoted by the suffix “s” (e.g., Q60s-, N11s-) are the side-chain resonances of Asn, Gln, and Arg.

**Figure 3:**
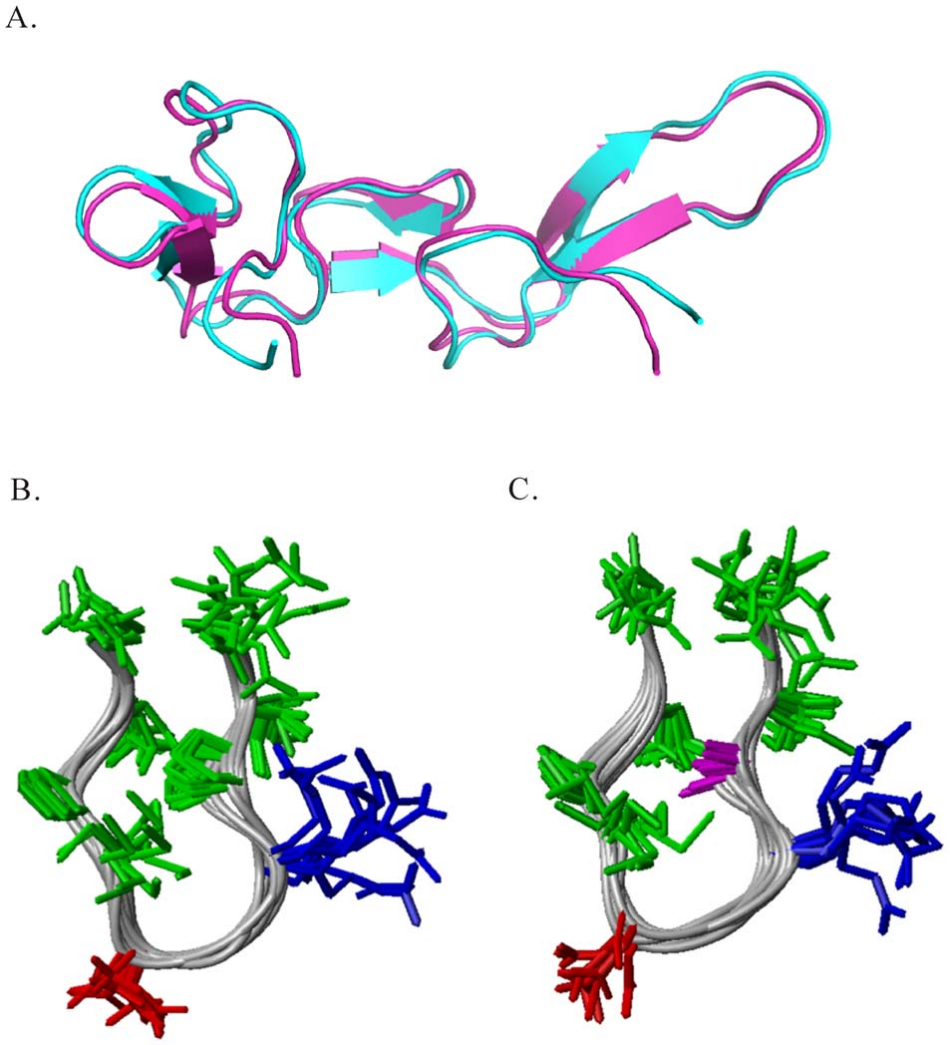
A structural comparison of Rho and its P48A mutant. (A) Superimpositions of the 3D structures of Rho (2PJF, cyan) and its P48A mutant (2PJI, light purple). Structural alignments of (B) the nine-residue RGD loop containing RI[P/A]RGDMPD sequences of Rho and (C) its P48A mutant. Ten of twenty Rho and its P48A mutant structures were aligned; the RMS deviations of the nine-residue backbone atoms of Rho and its P48A mutant were 0.551 and 0.475 Å, respectively. The side chains of A48, R49, D51, and others are shown in purple, blue, red, and green, respectively.

The superimposition of the ^15^N-HSQC spectra of Rho and its P48A mutant is shown in Figure 2. The chemical difference was calculated using the formula: 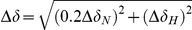. Chemical shift differences larger than 0.3 ppm were observed only for the residues close to mutation sites for R46, I47, R49, G50, D51, and D55, which were 0.52, 0.42, 0.57, 0.49, 0.91, and 0.38 ppm. Although there were chemical shifts of these residues, the amide strips from I47 to M52 of ^15^N-edited NOESY of Rho and its P48A mutant exhibited similar NOE patterns (Figure S3). Superimposing 3D structures of Rho and its P48A mutant showed that there was no difference in their overall structures (Figure 3A). Their RGDM motif, the binding sites for integrin, had a similar conformation: a type-I turn. Ten out of twenty of Rho and its P48A mutant structures were selected to align the nine-residue RGD loop, and the RMS deviations of the nine-residue backbone atoms of Rho and its P48A mutant were 0.55 and 0.40 Å, respectively (Figures 3B and 3C). This structural analysis showed that their RGD loops have the same backbone conformation.

### No structural differences between the integrin α5β1 complexes of Rho and the P48A mutant

**Figure 4:**
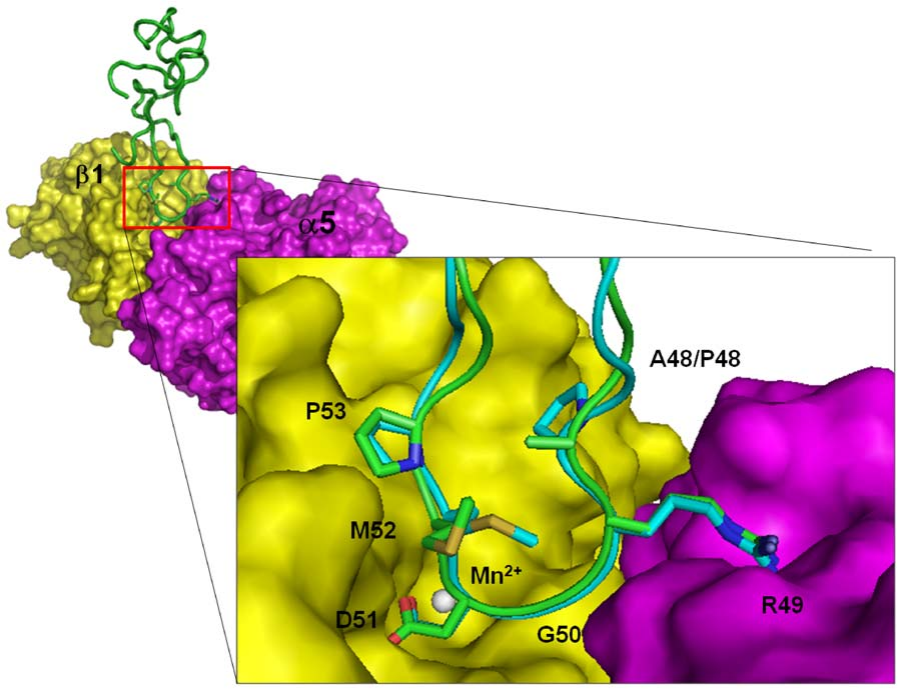
Docking models of Rho and its P48A mutant with integrin α5β1. Representation of Rho (cyan) and its P48A mutant (green) docked on the surface of modelling α5β1. The propeller domain of the α5 subunit and the βA domain of the β1 subunit are in purple and yellow, respectively. A snapshot of the interaction site is also shown. Side chains of the RGD motif of Rho and its P48A mutant are shown and labelled. Manganese ions at MIDAS are in white.

The dockings of Rho and the P48A mutant into integrin α5β1 were used to identify their integrin-interacting residues. The models of the integrin α5β1 complexes were built using HADDOCK 2.1 software [35]. The distance and hydrogen bond restraints were derived from the X-ray structure of integrin αvβ3 complexed with a cyclic RGD pentapeptide (PDB code 1L5G), and eight key interactions were found between integrin αvβ3 and the R and D residues [37]. According to sequence and structure alignments between integrins αvβ3 and α5β1, we identified the corresponding residues of integrin α5β1. For example, the R residue of the cyclic peptide forms a bidentate salt-bridge hydrogen bond with the D218 residue αv subunit, and its corresponding responding residue of the α5 subunit was the D227 residue. The carboxylate oxygen of the D residue of the cyclic peptide forms hydrogen bonds with the residues S121 and S123 of the β3 subunit, and the corresponding residues of the β1 subunit were residues S121 and S123. The other carboxyl oxygen of the D residue of the cyclic peptide forms hydrogen bonds with the residues Y122 and N215 of β3 subunit, and their corresponding residues of integrin β1 were Y122 and N213 (Figure 4). Using these restraints, we docked Rho and the P48A mutant to integrin α5β1, which, the analysis showed, resulted in the same number of contacts (Table S2). The key contacts—seven hydrogen bonds and two salt bridges between the R and D residues of the RGD motif and integrin α5β1—were the same. The total number of interactions between the R49 and D51 residues and integrin α5β1 were 41 and 49, respectively. In contrast, the P48 residue of Rho and the A48 residue of P48A mutant did not interact with integrin α5β1. This is consistent with our reported structures that both the A and P residues were located in the interior of the RGD loop (Figure 3C).

### Dynamics Differences between Rho and its P48A Mutant

**Figure 5:**
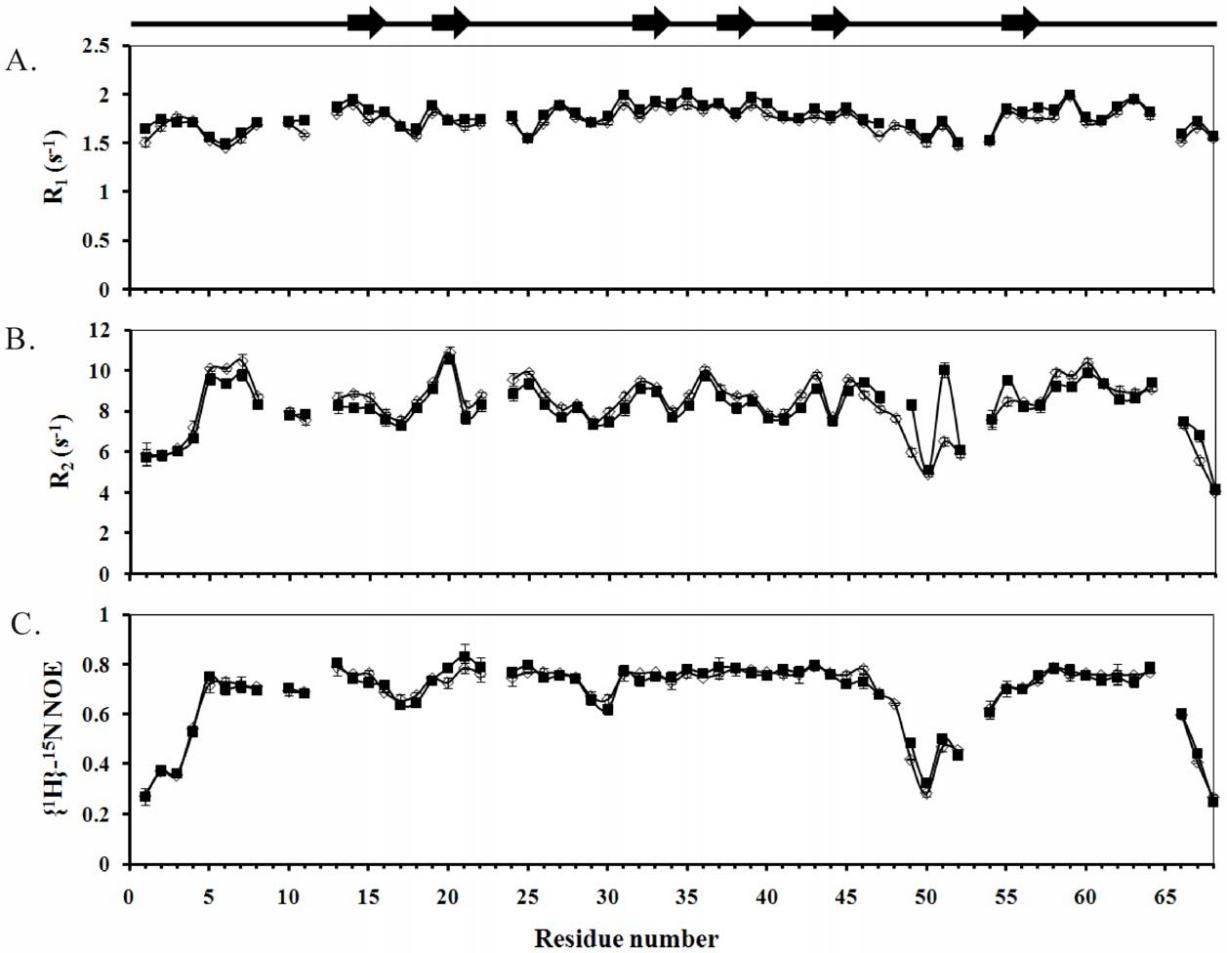
(A) A comparison of the relaxation parameters of Rho (▪) and its P48A mutant (□). ^15^N-*R_1_* with error. (B) ^15^N-*R_2_* with error. (C) ^1^H-^15^N steady-state NOE with error. These experiments were done using 600-MHz NMR. Gaps indicate the proline residues. The β-sheet secondary structure is shown in the top panel.

^1^H-^15^N correlated NMR spectroscopy was used to measure ^15^N *R_1_*, ^15^N *R_2_*, and ^1^H-^15^N NOE parameters for Rho and its P48A mutant. They were measured at 600 MHz and 700 MHz ^1^H to confirm the results. The NOE and *R_1_* relaxation parameters of Rho and its P48A mutant were similar throughout the sequence (Figure 5 and Figure S4). The major differences between Rho and the P48A mutant were found in the *R_2_* relaxation parameters of the R49 and D51 residues. Their *R_2_* values, measured at 600 MHz ^1^H, were 8.32±0.13 s^−1^ and 10.02±0.10 s^−1^ for Rho and 5.98±0.10 s^−1^ and 6.50±0.02 s^−1^ for the P48A mutant. They were consistent with the results measured at 700 MHz ^1^H, and the *R_2_* values for the Rho residues R49 and D51 were 39% and 54% higher than those of the P48A mutant (Table S3). The square of the generalized order parameter, S^2^, the effective internal correlation time, τ_e_, and a conformational exchange broadening parameter, R_ex_, for each backbone amide NH vector were determined using model-free formalism to compare their differences [34]. The optimized τ_m_ values of the P48A mutant was determined to be 6.38 ns, and its obtained diffusion tensor was fully asymmetric with D_//_/D_⊥_ = 1.25. They were very similar to the reported values of Rho and the measurement at 700 MHz ^1^H [27].

**Figure 6:**
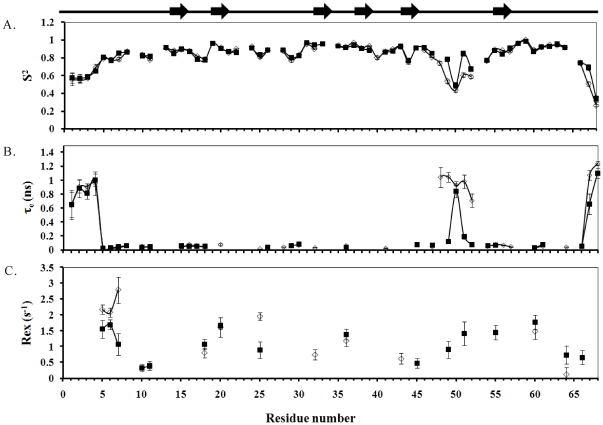
A comparison of the model-free parameters of Rho (▪) and its P48A mutant (□). (A) The square of the generalized order parameter S^2^. (B) The effective internal correlation time *τ*_e_. (C) The conformational exchange broadening parameter R_ex_. Gaps indicate the proline residues. The β-sheet secondary structure is shown in the top panel.

**Figure 7:**
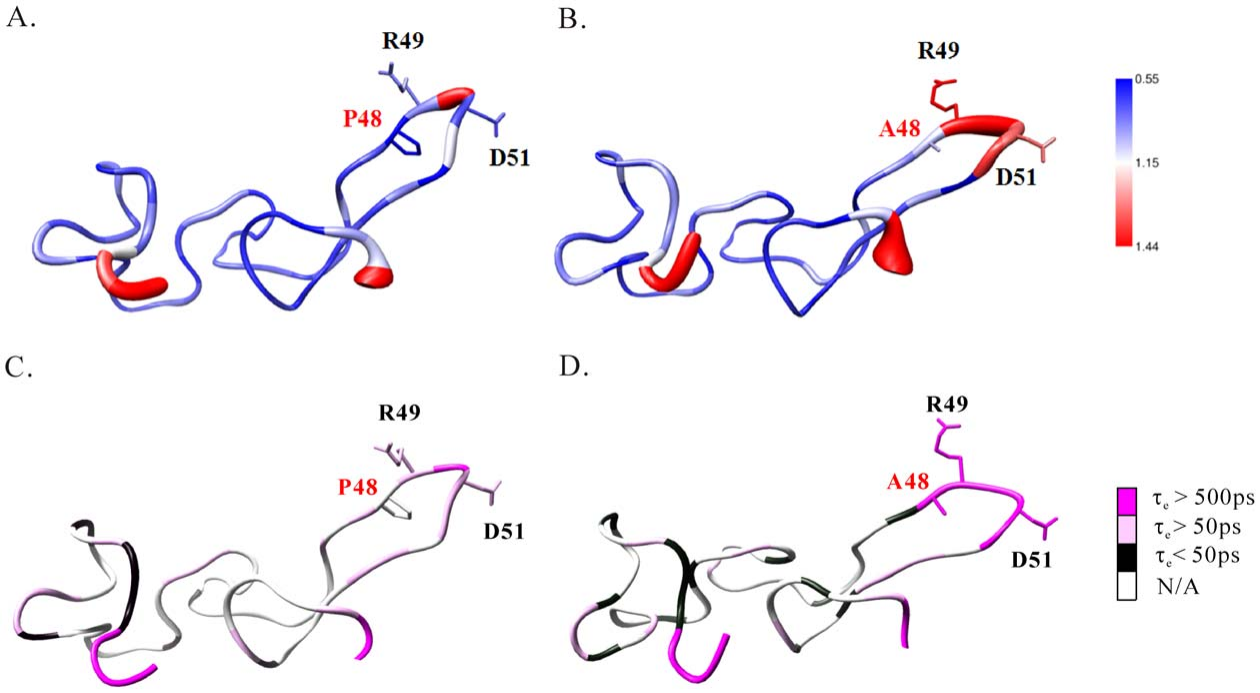
Representations of the dynamic properties of Rho and its P48A mutant. A sausage representation of the backbone dynamics of (A) Rho and (B) its P48A mutant. The diameter of the sausage is inversely proportional to the generalized order parameter of the corresponding residue. The side chains of P48/A48, R49, and D51 are shown. The magnitudes of the generalized order parameter are blue, white, and red. A representation of the effective internal correlation times of (C) Rho and (D) its P48A mutant. The residues containing the effective internal correlation times (*τ*_e_)>500 ps, >50 ps, and <50 ps are shown in dark pink, light pink, and black. The residues without relaxation data and the fitting model are shown in white.

The optimized values of S^2^, τ_e_, and R_ex_ of Rho and its P48A mutant were obtained from the measurements of 600 and 700 MHz ^1^H (Figure 6, Figure S5, Table 4, and Table S3). The observable S^2^ differences between Rho and its P48A mutant were found only in the RGD residues: the S^2^ values of the R49, G50, and D51 residues of the P48A mutant were 29%, 14%, and 28% lower than those of Rho. Their differences in RGD motif can be seen more clearly in the plots shown in Figures 7A and 7B. Furthermore, the major differences were found in τ_e_ and in the R_ex_ of R49 and D51: the R_ex_ values of R49 and D51 residues of Rho were 0.91 s^−1^ and 1.42 s^−1^, but we found no R_ex_ values for the P48A mutant residues. The τ_e_ values of R49 and D51 of Rho were 0.11 ns and 0.19 ns, and those of the P48A mutant were 1.04 ns and 0.98 (Table 4). The τ_e_ values of R49 and D51 residues of Rho were 9.5 and 5.1 times higher than those of the P48A mutant. The τ_e_ difference in the RGD motif between Rho and the P48A mutant can be seen clearly in the structural plot shown in Figures 7C and 7D. These results suggest that the flexibility and fast motion of the R49 and D51 residues on the ps/ns time scale may be important for binding integrin α5β1. It has been shown that the RGD loop and the C-terminal region of echistatin exhibit concerted motions [17]. In contrast, these regions of Rho did not exhibit concerted motions because no long-range NOEs between their backbone atoms were identified (Figure S2).

**Table 4:** Relaxation data and dynamic parameters of RGD-containing proteins.

	R_1_ (s^−1^)	R_2_ (s^−1^)	NOE	S^2^	τ_e_ (ns)	R_ex_ (s^−1^)
A48^P48A^ a	1.68±0.07	7.65±0.13	0.64±0.01	0.74±0.02	1.04±0.14	
R49^WT^ b	1.69±0.08	8.32±0.13	0.48±0.01	0.75±0.01	0.11±0.01	0.91±0.26
R49^P48A^ a	1.63±0.04	5.98±0.01	0.42±0.01	0.53±0.02	1.04±0.06	
R78^FN10^ c				0.46	3.25	
G50^WT^ b	1.55±0.02	5.09±0.03	0.32±0.02	0.50±0.03	0.84±0.08	
G50^P48A^ a	1.51±0.02	4.90±0.01	0.28±0.01	0.43±0.02	0.93±0.06	
G79^FN10^ c				0.39	2.75	
D51^WT^ b	1.72±0.03	10.03±0.02	0.50±0.02	0.85±0.01	0.19±0.03	1.42±0.37
D51^P48A^ a	1.68±0.02	6.50±0.02	0.47±0.02	0.61±0.02	0.98±0.09	
D80^FN10^ c				0.52	2.40	

a Rhodostomin P48A mutant from this study.

b Rhodostomin from Chen et al. [27].

c Dynamic properties of fibronectin type III domain reported by Carr et al. [52].

### Effect of N-terminal Residue Adjacent to the RGD Motif on the Inhibition of Integrin α5β1

To study the effect of the N-terminal residue of the RGD motif on the inhibition of integrin α5β1, we produced P48G, P48Y, P48F, P48W, P48L, and P48I mutants in *P. pastoris*. Compared with the P48A mutant, their inhibition of integrin α5β1 was 1.6, 5.3, 4.8, 3.3, 4.8, and 4.8 times lower (Table 5). The P48A and P48G mutants exhibited highest inhibitory activity. In contrast, the N-terminal residue containing the large hydrophobic amino acids was less inhibitory. These results suggest that proteins containing N-terminal alanine and glycine residues adjacent to the RGD motif may increase their binding to integrin α5β1.

**Table 5:** Summary of inhibition of K562 cell adhesion to fibronectin by Rho and its mutants.

Protein						IC50 (nM)	
**P**	R	G	D	M	P	231.4	±91.8
**A**	R	G	D	M	P	59.0	±28.4
**G**	R	G	D	M	P	92.2	±29.1
**Y**	R	G	D	M	P	310.6	±56.9
**F**	R	G	D	M	P	282.1	±43.2
**W**	R	G	D	M	P	193.2	±91.1
**L**	R	G	D	M	P	283.4	±126.3
**I**	R	G	D	M	P	281.7	±71.4

## Discussion

The residues flanking the RGD motif of RGD-containing proteins affect their binding specificities and affinities on integrins [7], [10], [22], [23], [24], [25], [40]. In the present study, we showed that the replacement of N-terminal proline with alanine or glycine adjacent to the RGD motif in Rho increased their binding affinity to integrin α5β1; similar results were found with Rho P48A mutants having different C-terminal residues adjacent to the RGD motif. No structural differences between Rho and its P48A mutant or between their integrin α5β1 complexes were found. The only difference was found in the backbone dynamics of the RGD residues. The proline-to-alanine mutation increased the flexibility of the RGD residues and the fast motion of the R and D residues on the ps-ns timescale. These results showed that the N-terminal proline residue adjacent to the RGD motif of Rho affects its function and dynamics, but does not affect the conformation of the RGD motif, which suggests that the flexibility and the motions of the RGD residues may be important for their interaction with integrin α5β1.

Proline residue is the only common imino acid in proteins with a bulky pyrrolidine ring that restricts the conformational range of its adjacent residues. The lack of a proton on the imino nitrogen of proline blocks the hydrogen-bond formation required for α-helix and β-sheet secondary structures, and thus disrupts the propagation of neighbouring secondary structures. Therefore, it is commonly found in the turn and loop structures of proteins [41], [42]. The occurrence of a proline residue in a protein sequence often has a strong influence on the protein's stability, structure, and function [42], [43], [44], [45]. The effects of proline residue are diverse, and they depend on their neighbouring residues and structural contexts [42], [43], [44], [45]. For example, the replacement of proline on lysozyme and lambda repressor caused protein instability [42], [44]. In contrast, the replacement of proline on the α-subunit of tryptophan synthase and staphylococcal nuclease increased their protein stability [46], [47], [48]. In some cases, the effect of proline replacement on protein stability can be only marginal [49], [50]. The proline residue is also important in many structural elements, such as an N-terminal cap residue in the α-helix, a terminal residue in the α-helix, and a corner residue in the β-turn structure. Our findings are an example of a role for N-terminal amino acid adjacent to the RGD motif in determining the activity of RGD proteins. We found that the mutation of proline to alanine or glycine on Rho increased their binding affinity to integrin α5β1. The N-terminal proline residue adjacent to the RGD motif may provide an unfavourable environment for inhibiting integrin α5β1. The analyses of the primary sequences of disintegrins showed that >98% of their RGD loops have an **A**RGD amino acid sequence [26]. The RGD loop sequences of natural integrin α5β1 ligands, such as fibronectin, osteopontin, and thrombospondins, are **G**RGDS, **G**RGDS, and **G**RGD(A/I) [1]. These results suggest that integrin α5β1 prefers its ligands to contain **(G/A)**RGD but not **P**RGD sequences for binding.

The synergy between structure and dynamics is essential to the function of biological complexes. We found that the effect of the N-terminal proline residue of the RGD motif in Rho on reducing its binding affinity to integrin α5β1 may be due to its effect on the dynamic properties of the RGD motif. The proline effect is commonly attributed to the limitation of its backbone entropy. The cis-trans isomerisation of the proline peptide bond is also responsible for the activities of many proteins and peptides [51]. Proline isomerization can induce conformational heterogeneity and control the binding and function of globular proteins. In the present study, we found that the proline residue of P48A mutants did not exhibit cis-trans isomerisation and had only a trans conformation. The P48A mutation caused a decrease in the *R_2_* values of the R and D residues, which resulted in a decrease of the rigidity, the disappearance of conformational exchange, and the increase of fast motion on the ps-ns timescale of the RGD motif. The RGD motif, the integrin-binding site, of P48A mutant is clearly more flexible on the ps-ns timescale than on that of Rho, as indicated by both the dramatically lower-order parameters and much larger effective internal correlation times. This effect may facilitate the P48A mutants to interact with integrin α5β1. This is consistent with the dynamic features of the RGD motif in fibronectin, which preferentially binds to integrin α5β1 [52]. The conformational freedom of the RGD loop in P48A mutant results in high flexibility and solvent exposure of this loop, which may be responsible for its fast recognition and fitting to integrin α5β1 [17], [34], [53], [54]. However, a detailed investigation of the individual integrin α5β1 ligands may be required.

In conclusion, we found that the N-terminal proline residue adjacent to the RGD motif affects the function and dynamic properties of the RGD motif, which shows that the dynamic properties of the RGD motif in RGD-containing proteins may be important for integrin recognition. Our functional analysis also showed that the integrin α5β1 ligands prefer to have N-terminal residue that contains either glycine or alanine amino acid. These results provide important dynamic information for designing potent RGD mimetics and serve as the basis for exploring the structure and the functional relationships of RGD-binding integrins and their ligands.

## Supporting Information

Figure S1 **Mass spectra of recombinant Rho variants.**
(TIF)

Figure S2 **Map of the NOE connectivities of detected in Rho (A) and its P48A mutant (B).** NOEs involving the sidechain resonances are plotted below the diagonal, and those involving only mainchain resonances are plotted above the diagonal.
(TIF)

Figure S3 **Amide strips from I47 to M52 of Rho (A) and its P48A mutant (B) at pH 6.0.** The dNN (i, i +1) and dαN (i, i +1) NOE connectivities are shown.
(TIF)

Figure S4 **Comparison of the relaxation parameters of Rho (▪) and its P48A mutant (□).** ^15^N *R_1_* with error (A). ^15^N *R_2_* with error (B).^1^H-^15^N steady-state NOE with error (C) These experiments were acquired using 700 MHz NMR.
(TIF)

Figure S5 **Comparison of model-free parameters of Rho (▪) and its P48A mutant (Δ).** Generalized order parameters S^2^, *τ*_e_, and R_ex_ (calculated from 700 MHz relaxation data). Gaps indicate the proline residues, and the β-sheet secondary structure is shown. Comparison of internal timescale parameters, *τ*_e_, of Rho and its P48A Mutant (B). Only some fitting models resulted in a τ_e_ term. Comparison of the conformational exchange terms, R_ex_, for Rho and its P48A mutant (C). Only some fitting models resulted in an R_ex_ term.
(TIF)

Table S1 **Molecular weights of recombinant Rho variants.**
(DOC)

Table S2 **Summary of the interactions between protein and integrin.**
(DOC)

Table S3 **Relaxation data and dynamic parameters of Rho and P48A mutant (700 MHz).**
(DOC)
