# Supplementary figures and images for: Effect of P to A Mutation of the N-Terminal Residue Adjacent to the Rgd Motif on Rhodostomin: Importance of Dynamics in Integrin Recognition
Source: PLoS One. 2012 Jan 4;7(1):e28833. doi: 10.1371/journal.pone.0028833 (PMC3251565; doi:10.1371/journal.pone.0028833)

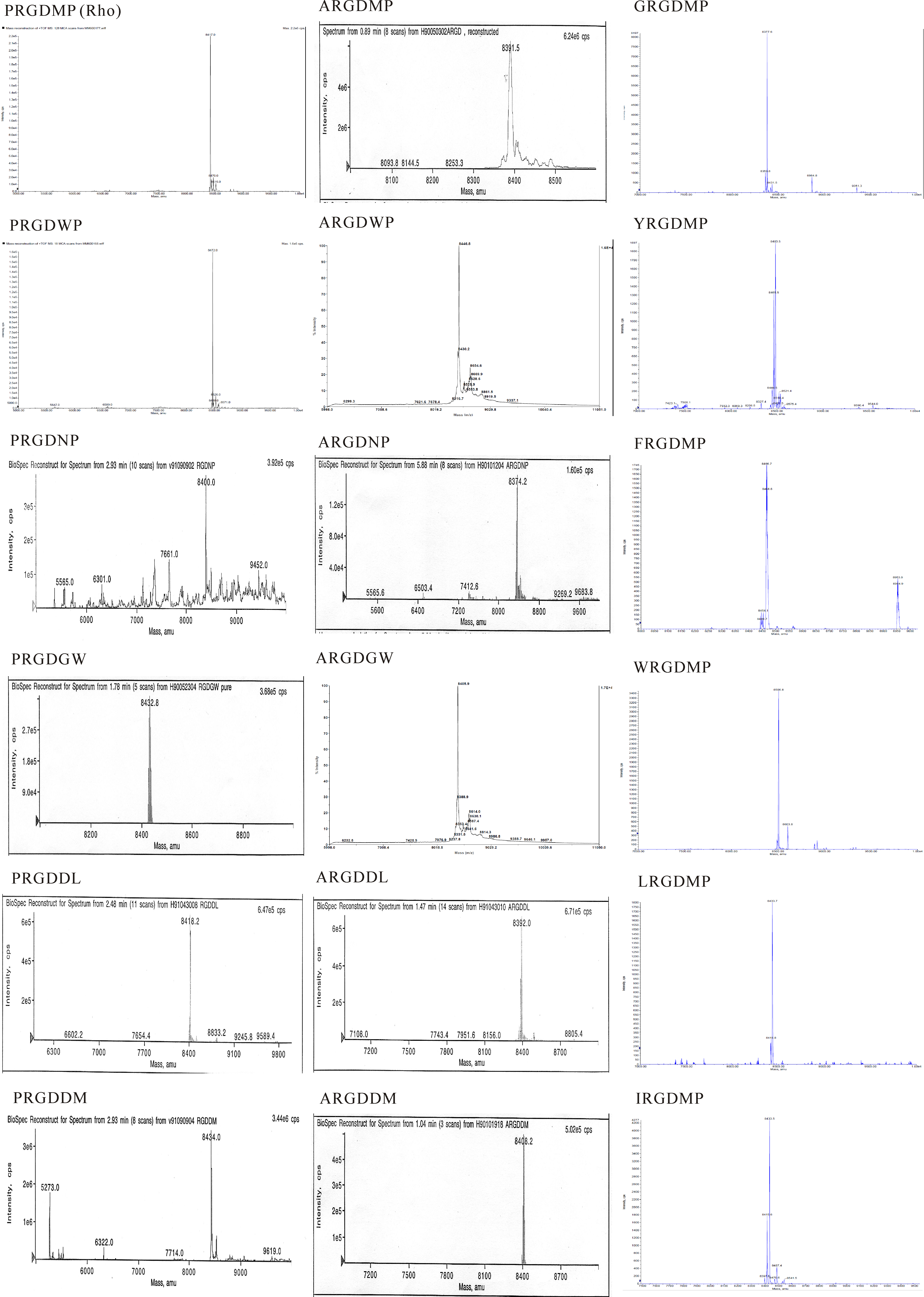

Supplement: Figure S1 — Mass spectra of recombinant Rho variants. (TIF) [file pone.0028833.s001.tif]

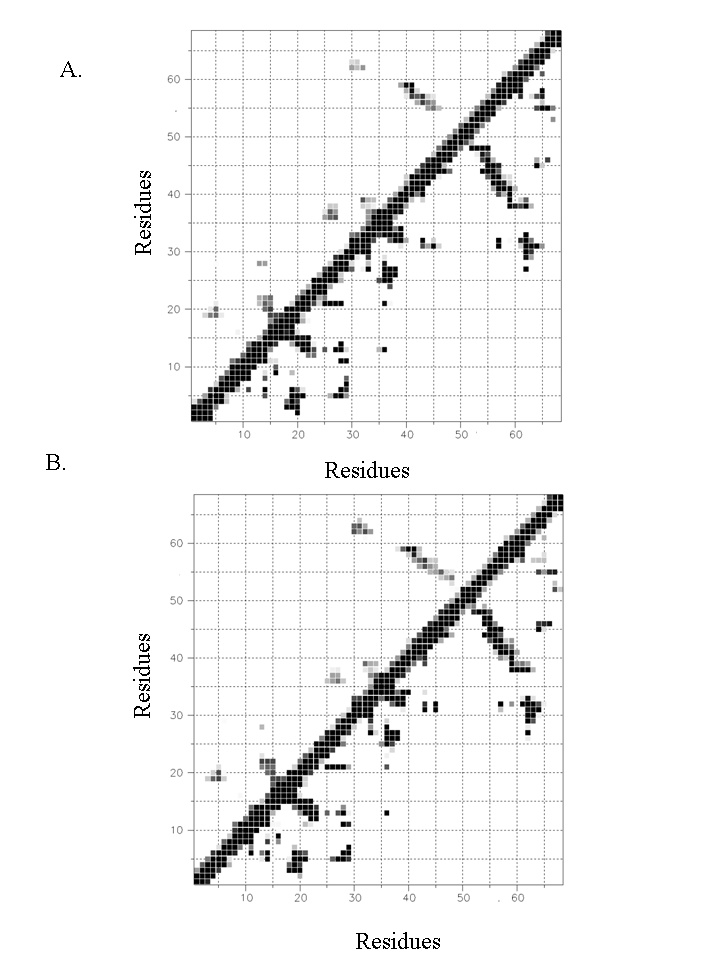

Supplement: Figure S2 — Map of the NOE connectivities of detected in Rho (A) and its P48A mutant (B). NOEs involving the sidechain resonances are plotted below the diagonal, and those involving only mainchain resonances are plotted above the diagonal. (TIF) [file pone.0028833.s002.tif]

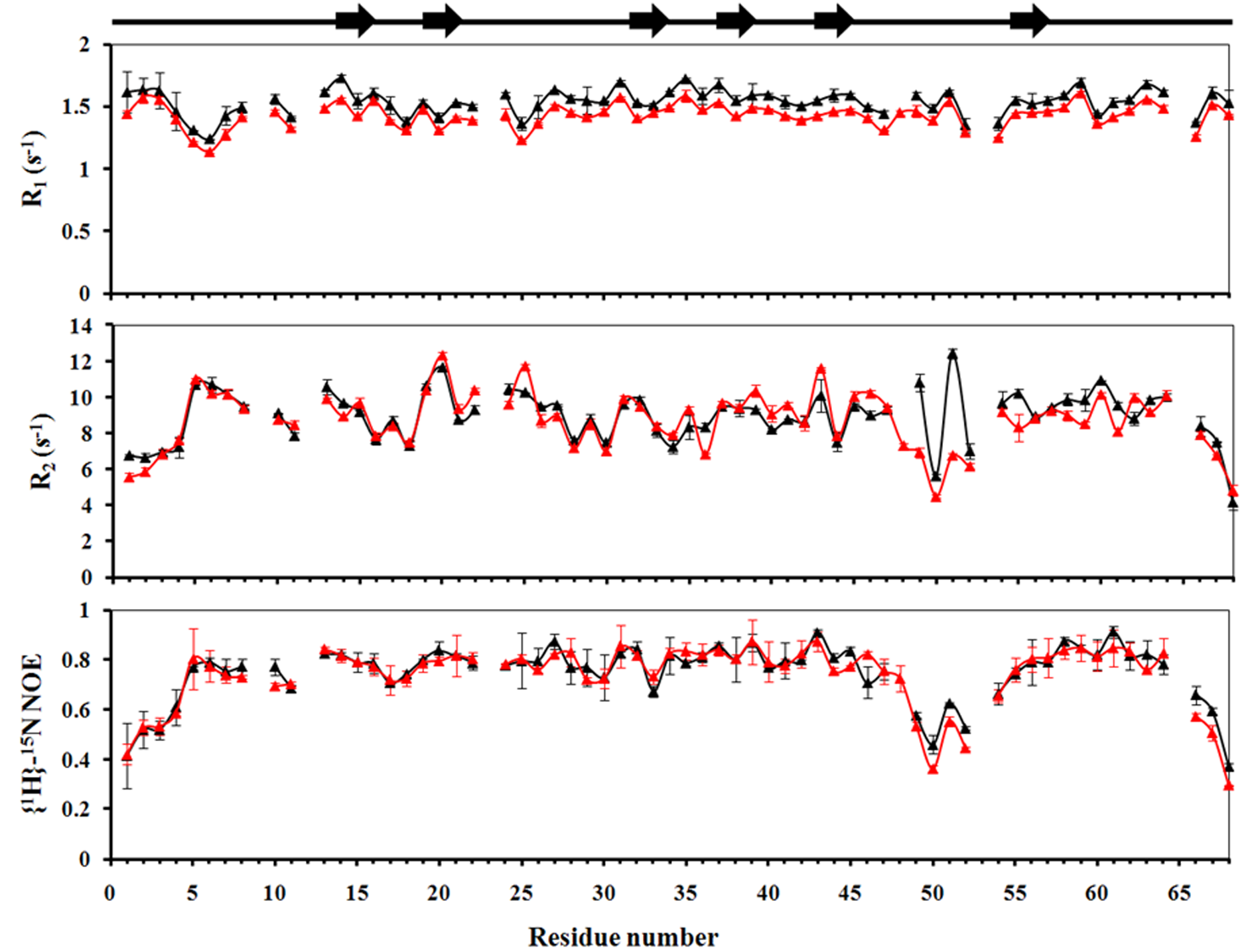

Supplement: Figure S4 — Comparison of the relaxation parameters of Rho (▪) and its P48A mutant (□). 15N R1 with error (A). 15N R2 with error (B).1H-15N steady-state NOE with error (C) These experiments were acquired using 700 MHz NMR. (TIF) [file pone.0028833.s004.tif]

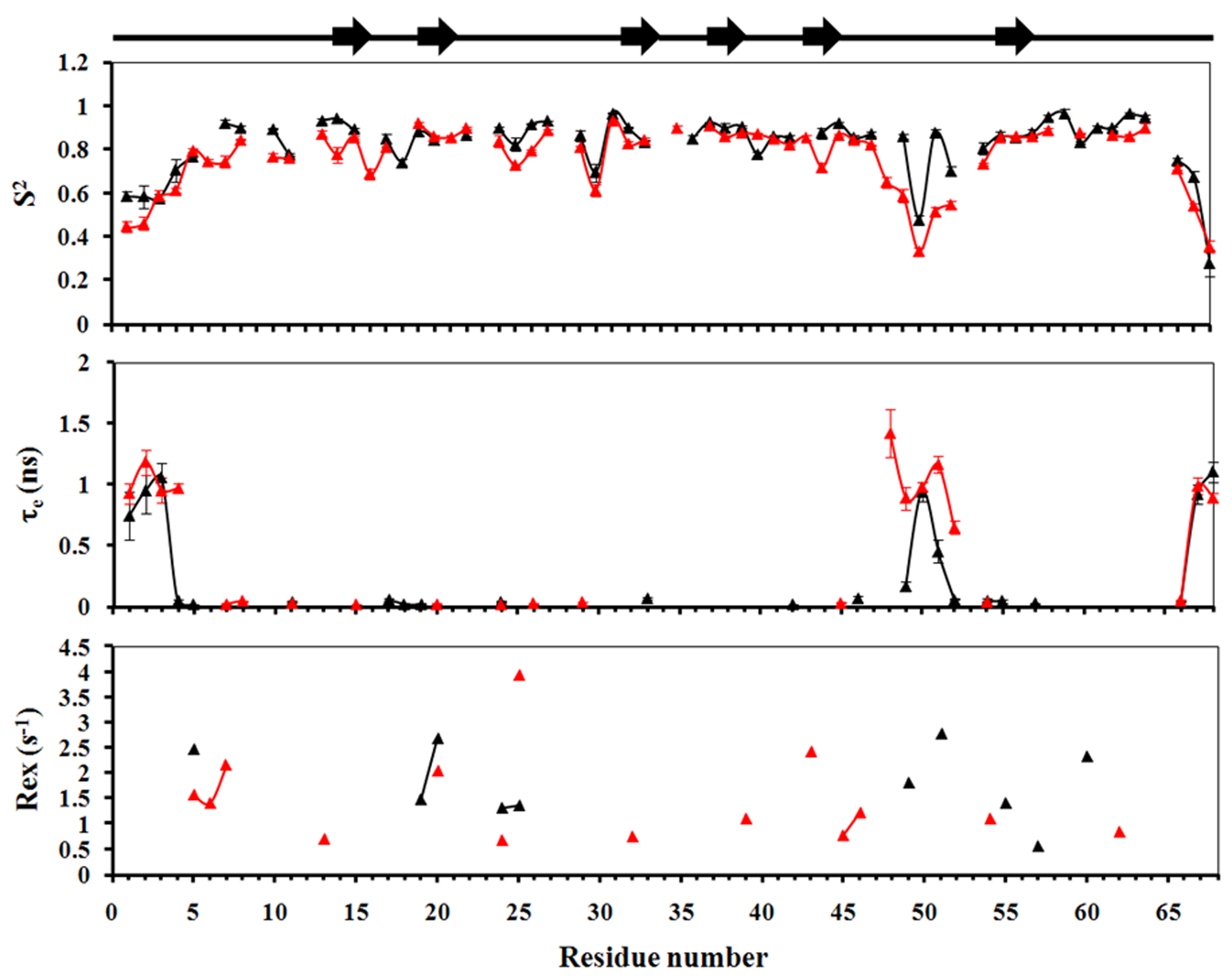

Supplement: Figure S5 — Comparison of model-free parameters of Rho (▪) and its P48A mutant (Δ). Generalized order parameters S2, τ e, and Rex (calculated from 700 MHz relaxation data). Gaps indicate the proline residues, and the β-sheet secondary structure is shown. Comparison of internal timescale parameters, τ e, of Rho and its P48A Mutant (B). Only some fitting models resulted in a τe term. Comparison of the conformational exchange terms, Rex, for Rho and its P48A mutant (C). Only some fitting models resulted in an Rex term. (TIF) [file pone.0028833.s005.tif]
