# Supplementary material for: Effect of P to A Mutation of the N-Terminal Residue Adjacent to the Rgd Motif on Rhodostomin: Importance of Dynamics in Integrin Recognition
Source: PLoS One. 2012 Jan 4;7(1):e28833. doi: 10.1371/journal.pone.0028833 (PMC3251565; doi:10.1371/journal.pone.0028833)
Supplement: Table S1 — Molecular weights of recombinant Rho variants. (DOC) [file pone.0028833.s006.doc]

| **Sequence of Rho mutants** | | | | | | **M.W.** | | |
| --- | --- | --- | --- | --- | --- | --- | --- | --- |
| **48** | **49** | **50** | **51** | **52** | **53** | **Calculated** | **Experimental** | **Deviation** |
| P | R | G | D | M | P | 8417.3 | 8417.0 | -0.3 |
| P | R | G | D | **W** | P | 8472.4 | 8472.0 | -0.4 |
| P | R | G | D | **N** | P | 8400.3 | 8400.0 | -0.3 |
| P | R | G | D | G | W | 8432.4 | 8432.8 | 0.4 |
| P | R | G | D | D | L | 8417.3 | 8418.2 | 0.9 |
| P | R | G | D | **D** | **M** | 8434.9 | 8434.0 | -0.9 |
| A | R | G | D | M | P | 8391.4 | 8391.5 | 0.1 |
| A | R | G | D | **W** | P | 8446.4 | 8446.8 | 0.4 |
| **A** | R | G | D | **N** | P | 8374.3 | 8374.2 | -0.1 |
| **A** | R | G | D | **G** | **W** | 8406.4 | 8405.9 | -0.5 |
| **A** | R | G | D | **D** | **L** | 8391.3 | 8392.0 | 0.7 |
| **A** | R | G | D | **D** | **M** | 8409.1 | 8408.2 | -0.9 |
| **G** | R | G | D | M | P | 8377.4 | 8377.6 | 0.2 |
| **Y** | R | G | D | M | P | 8483.6 | 8483.5 | -0.1 |
| **F** | R | G | D | M | P | 8467.5 | 8466.7 | -0.8 |
| **W** | R | G | D | M | P | 8506.5 | 8506.8 | 0.3 |
| **L** | R | G | D | M | P | 8433.5 | 8433.7 | 0.2 |
| **I** | R | G | D | M | P | 8433.5 | 8433.5 | 0.0 |

Table S1. Molecular weights of recombinant Rho variants
