# Supplementary material for: Effect of P to A Mutation of the N-Terminal Residue Adjacent to the Rgd Motif on Rhodostomin: Importance of Dynamics in Integrin Recognition
Source: PLoS One. 2012 Jan 4;7(1):e28833. doi: 10.1371/journal.pone.0028833 (PMC3251565; doi:10.1371/journal.pone.0028833)
Supplement: Table S2 — Summary of the interactions between protein and integrin. (DOC) [file pone.0028833.s007.doc]

Table S2. Summary of the interactions between protein and integrin

| Protein | Residue | | Numbers of interaction | | | |
| --- | --- | --- | --- | --- | --- | --- |
|  |  |  | Integrin **α5** | | Integrin **β1** | |
| Rho | P48 | Backbone | 0 |  | 0.07 | ± 0.25 |
|  |  | Sidechain | 0 |  | 0.13 | ± 0.51 |
|  | R49 | Backbone | 3.67 | ± 1.39 | 0.07 | ± 0.25 |
|  |  | Sidechain | 38.87 | ± 3.46 | 0 |  |
|  | G50 | Backbone | 0.87 | ± 1.06 | 16.87 | ± 4.42 |
|  |  | Sidechain | 0 |  | 0 |  |
|  | D51 | Backbone | 0 |  | 8.13 | ± 2.19 |
|  |  | Sidechain | 0 |  | 41.00 | ± 4.50 |
| P48A | A48 | Backbone | 0 |  | 0 |  |
|  |  | Sidechain | 0 |  | 0 |  |
|  | R49 | Backbone | 3.42 | ± 1.34 | 0.28 | ± 1.06 |
|  |  | Sidechain | 38.21 | ± 3.61 | 0 |  |
|  | G50 | Backbone | 0.42 | ± 0.75 | 12.28 | ± 2.01 |
|  |  | Sidechain | 0 |  | 0 |  |
|  | D51 | Backbone | 0 |  | 8.57 | ± 1.55 |
|  |  | Sidechain | 0 |  | 42.42 | ± 3.45 |
