# Supplementary material for: Effect of P to A Mutation of the N-Terminal Residue Adjacent to the Rgd Motif on Rhodostomin: Importance of Dynamics in Integrin Recognition
Source: PLoS One. 2012 Jan 4;7(1):e28833. doi: 10.1371/journal.pone.0028833 (PMC3251565; doi:10.1371/journal.pone.0028833)
Supplement: Table S3 — Relaxation data and dynamic parameters of Rho and P48A mutant (700 MHz). (DOC) [file pone.0028833.s008.doc]

Table S3. Relaxation data and dynamic parameters of Rho and P48A mutant (700MHz)

|  | R_1_ (s^-1^) | R_2_ (s^-1^) | NOE | S^2^ | τ_e_ (ns) | R_ex_ (s^-1^) |
| --- | --- | --- | --- | --- | --- | --- |
| A48^P48A^a | 1.45±0.02 | 7.28±0.12 | 0.72±0.05 | 0.65±0.02 | 1.42±0.49 |  |
| R49^WT^c | 1.59±0.02 | 10.80±0.46 | 0.57±0.01 | 0.86±0.01 | 0.16±0.03 | 1.81±0.48 |
| R49^P48A^a | 1.46±0.04 | 6.91±0.25 | 0.53±0.01 | 0.58±0.03 | 0.88±0.09 |  |
| G50^WT^c | 1.49±0.03 | 5.63±0.11 | 0.45±0.04 | 0.48±0.02 | 0.93±0.07 |  |
| G50^P48A^a | 1.38±0.03 | 4.48±0.10 | 0.36±0.01 | 0.33±0.02 | 0.98±0.03 |  |
| D51^WT^c | 1.62±0.01 | 12.42±0.28 | 0.62±0.01 | 0.87±0.01 | 0.44±0.09 | 2.78±0.29 |
| D51^P48A^a | 1.53±0.03 | 6.74±0.15 | 0.55±0.02 | 0.52±0.02 | 1.16±0.06 |  |

^a^Rhodotomin P48A mutant from this study.

^b^Dynamical properties of echistatin reported by Chen et al. (1994).

^c^Rhodostomin from this study.

^d^Dynamical properties of fibronectin type III domain reported by Chen et al. (1997)
